# A Colourful Clock

**DOI:** 10.1371/journal.pbio.1002160

**Published:** 2015-05-21

**Authors:** Hester C. van Diepen, Russell G. Foster, Johanna H. Meijer

**Affiliations:** 1 Laboratory for Neurophysiology, Department of Molecular Cell Biology, Leiden University medical School, Leiden, The Netherlands; 2 Nuffield Laboratory of Ophthalmology; Sleep and Circadian Neuroscience Institute (SCNi) Nuffield Department of Clinical Neurosciences, University of Oxford, John Radcliffe Hospital, Oxford, United Kingdom

## Abstract

Circadian rhythms are an essential property of life on Earth. In mammals, these rhythms are coordinated by a small set of neurons, located in the suprachiasmatic nuclei (SCN). The environmental light/dark cycle synchronizes (entrains) the SCN via a distinct pathway, originating in a subset of photosensitive retinal ganglion cells (pRGCs) that utilize the photopigment melanopsin (OPN4). The pRGCs are also innervated by rods and cones and, so, are both endogenously and exogenously light sensitive. Accumulating evidence has shown that the circadian system is sensitive to ultraviolet (UV), blue, and green wavelengths of light. However, it was unclear whether colour perception itself can help entrain the SCN. By utilizing both behavioural and electrophysiological recording techniques, Walmsley and colleagues show that multiple photic channels interact and enhance the capacity of the SCN to synchronize to the environmental cycle. Thus, entrainment of the circadian system combines both environmental irradiance and colour information to ensure that internal and external time are appropriately aligned.

## Abstract 2

New evidence reveals that entrainment of the circadian clock combines both environmental irradiance and color information to ensure that internal and external time are appropriately aligned.

Light is sensed by three classes of retinal photoreceptors. In the outer retina, light is detected by rod and cone photoreceptors; in the inner retina a small number of photosensitive retinal ganglion cells (pRGCs) express the photopigment melanopsin, which confers photosensitivity to these neurons. The signals derived from the various photoreceptors are important for visual and nonvisual tasks. The generation of visual images is primarily a function of the classical rod and cone photoreceptors, while the classical photoreceptors together with melanopsin are involved in nonvisual tasks, such as pupillary reflexes and the synchronization of our circadian clock to the environmental light-dark cycle. The discovery of non-rod, non-cone photoreceptors [1] and the demonstration that they utilize the photopigment melanopsin [2] led to the general and unfortunate notion that melanopsin is the major—if not the only—photopigment that contributes to photoentrainment and that this sensory task is monochromatic, with no role for colour discrimination. The article of Walmsley et al. [3] addresses this misconception and presents evidence for a role for colour detection in photoentrainment.

These findings are in accordance with recent publications indicating that not only melanopsin but also other photopigments contribute to entrainment [4–9]. The consensus from these studies is that rods are most important for photoentrainment at low light intensities; cone photoreceptors transduce light information to the suprachiasmatic nuclei (SCN) at intermediate and high irradiances and are able to detect sudden changes in light intensity, whilst melanopsin detects light at high irradiances and may be of specific importance for the integration of light information over longer periods of time.

While it is true that the different photoreceptors are sensitive across a range of different light intensities, they are also maximally sensitive to different colours or wavelengths of light, and as a result, each class of photoreceptor has a different peak sensitivity. Rod photoreceptors have their peak sensitivity at 498 nm light (which would appear to us as green), melanopsin is maximally sensitive to 480 nm (blue) light, and most mammals express two distinct classes of cone photoreceptors, which in the majority of rodents are maximally sensitive to approximately 360 nm (UV) and approximately508 nm (green) light respectively. As a consequence, the different photoreceptive systems not only show differences in their absolute sensitivities, but in addition, they are differentially stimulated by different wavelengths of light. Theoretically, this characteristic difference in the spectral sensitivity of the photoreceptors could add to the detection of light intensities over the day-night cycle and thereby to the capacity of the SCN to adjust to it. Such a possibility was first suggested by Foster and colleagues [10] and shown for fish by Pauers and colleagues [11].

Walmsley et al. make use of a sophisticated experimental design to show the functional role of colour for the circadian system. Environmental light measurements were performed in Manchester, which lies 53 degrees north of the equator, as a function of solar angle relative to the horizon. Measurements were performed between August and October of 2005. The spectral measurements showed a reduction of irradiance and an increasing amount of short-wavelength light during twilight when the sun is below the horizon (Fig 1). This is a consequence of the differential scattering of shorter wavelengths of light by particles in the atmosphere and filtering of long wavelength light by the Chappuis band of the ozone layer. Based on the known spectral sensitivities of the short- and medium-wavelength—sensitive cone opsins, the excitation of the two pigments at different solar angles was calculated. Relative to the medium-wavelength—sensitive cone opsin, excitation of the short-wavelength—sensitive cone opsin decreases with increasing elevation of the sun above the horizon. The spectral composition of light reaching the earth shows less day-to-day variability in spectral composition than in irradiance, and thus, it may have a high predictive value about the position of the sun, as originally predicted by Foster (e.g., 2001).

**Fig 1:**
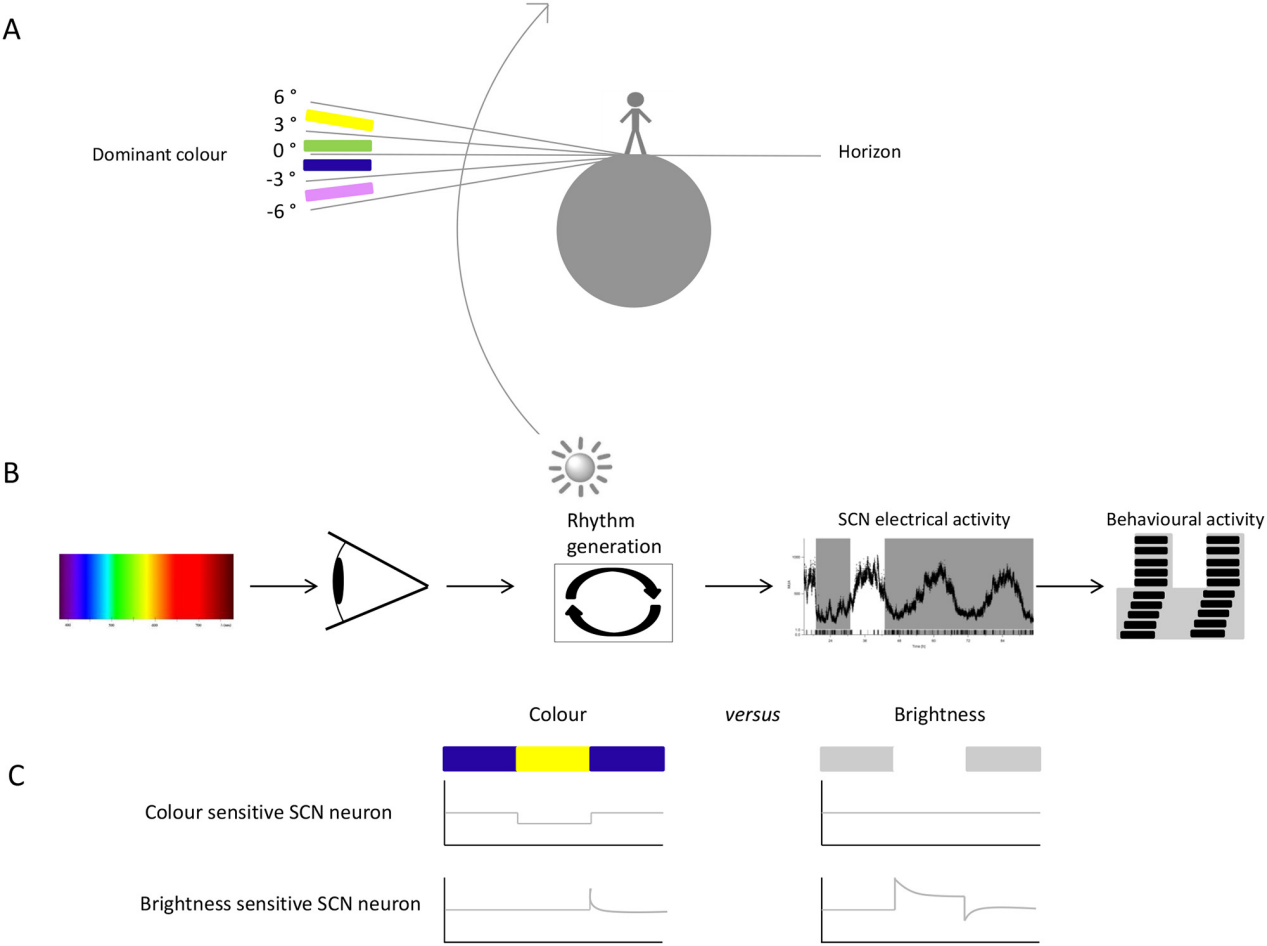
Colour detection by the circadian system. Colour detection by the circadian system. (A) Spectral changes in light reaching the earth during twilight. At negative solar angles, short wavelength light is dominant, while at positive solar angles, long wavelength light is dominant. (B) Schematic overview of light signalling to the SCN resulting in entrainment to a light-dark cycle. Light is the main entraining signal that adjusts the endogenous period length to the day-night cycle. Electrical activity of SCN neurons is the main output signal of the SCN, which leads to temporal regulation of behavioural activity. (C) Schematic depiction of two types of light-responsive neurons observed in the SCN as shown by Walmsley and colleagues: the colour-sensitive neuron (upper traces) and the brightness-sensitive neurons (lower traces). *Image credit*: *Hester van Diepen*.

The information about changes in spectral composition of light over the day were used to simulate twilight in laboratory conditions to study whether mice make use of these changes in colour as an estimation of the time of the day. Electrophysiological recordings from SCN neurons revealed that a subpopulation of light-responsive neurons is sensitive to changes in the spectral composition of daylight. These neurons were detected based on the presence of a response to changes in spectral composition of the light source, consisting of three light-emitting diodes (LEDs) with narrow band emittance at 365 nm, 460 nm, and 600 nm. These wavelengths maximally stimulate the short-wavelength, UV sensitive cone, melanopsin, and a red knock-in cone that substitutes the normal green cone and enhances discrimination between photoreceptors.

In addition to being sensitive to spectral composition changes, some neurons showed colour-opponency in response to selective activation of short-wavelength—sensitive opsins versus long-wavelength—sensitive opsins or vice versa (Fig 1). Cone photoreceptors display colour-opponency, most likely by combining signals from separate classes of cone photoreceptors in an opposing way [12,13]. The SCN may make use of this antagonistic effect by determination of the relative activation of the cone photoreceptors to various wavelengths of light. Since the two classes of photoreceptors in the mammalian retina are specifically sensitive to short-wavelength and long-wavelength light, the blue-yellow colour discrimination is a reliable way in which the SCN can detect transitions from twilight to daylight. In fact, behavioural experiments in mice showed that changes in colour are required for appropriate biological timing with respect to the solar cycle.

It is of utmost importance that the SCN is appropriately aligned with the environmental light-dark cycle. In rodents, the SCN consists of about 20,000 cell autonomous oscillators that are capable of producing circadian rhythms with a period deviating slightly from 24 hours. For proper function, the cells have to be mutually synchronized, and as an ensemble they should synchronize to the environmental cycle. Direct retinal input to the SCN, via the retinohypothaloamic tract (RHT), originates exclusively from pRGCs [14]. The pRGCs can be activated by rod and cone photoreceptors via synaptic connections to the outer retina [15]. Upon activation by light, photoreceptors undergo a transformational change from the inactive state to the active state, which results in a signalling cascade that ultimately leads to the generation of action potentials in the retinal ganglion cells and in the optic tract. The initial response of the classical photoreceptors is a hyperpolarization, while the conformational change of melanopsin leads to a depolarization. The present view, emerging from the various studies, is that light information reaches the SCN via all retinal photoreceptive systems. The ability of SCN neurons to not only determine the amount of light but also the wavelength of light by comparison of the relative activation of the different photoreceptors provides the SCN with additional information. The detection of changes in spectral composition may be an additive way to detect the time of the day-night cycle, as compared to irradiance detection alone. The amount of light perceived by the SCN can vary over the day, caused by covering of the sun with clouds or hiding of a mammal in its burrow [16]. The spectral composition of light during the lower light intensity time point will not change. Therefore, this perception system provides a refinement in the ability of the SCN to estimate time of day, which would not have been possible by the estimation of irradiance per se. As at least 90% of mammalian species can discriminate colour on the basis of at least two classes of cone opsins [17], it would be interesting to investigate to what degree other mammals also make use of colour to tell time of day.

## Abbreviations

LEDs: light-emitting diodes
pRGCs: photosensitive retinal ganglion cells
RHT: retinohypothalamic tract
SCN: suprachiasmatic nuclei
UV: ultraviolet
